# Digital Vibroacoustic stimulation for Insomnia in Adults With PTSD in a War Zone: A Feasibility Study

**DOI:** 10.1192/j.eurpsy.2026.11881

**Published:** 2026-07-31

**Authors:** U. Katz, S. Grinapol, R. Hazani, A. Paz, N. Strasberg, H. Shehade, S. Hevron, G. Zalsman

**Affiliations:** 1 Geha Mental Health Center, Petah Tikva; 2 Maale Hacarmel Mental Health Center, tel aviv, Israel

## Abstract

**Introduction:**

Insomnia is highly prevalent among individuals with posttraumatic stress disorder (PTSD) and is considered a hallmark symptom. Effective treatment of insomnia may improve both sleep and PTSD outcomes. Cognitive behavioral therapy for insomnia (CBT-I) is the recommended first-line treatment, but its availability is limited, underscoring the need for scalable digital alternatives

**Objectives:**

To evaluate the feasibility and efficacy of *TuneMe*, a personalized digital vibroacoustic intervention, for reducing insomnia symptoms in adults with PTSD in a war zone setting.

**Methods:**

This was a prospective, single-arm, open-label feasibility study conducted at Geha Mental Health Center, Israel. Thirty adults (aged 18–65 years) with PTSD and comorbid insomnia were recruited. All patients developed PTSD in a war zone setting over the last two years. Participants used the *TuneMe* application nightly, which produces personalized auditory stimulation based on real-time voice analysis and physiological monitoring. All patients completed the 7-week intervention.

The primary outcomes were changes in Insomnia Severity Index (ISI) and PTSD Checklist for DSM-5 (PCL-5) scores from baseline to week 7. Secondary outcomes included physiological stress, assessed with the Garmin stress index derived from heart rate variability.

**Results:**

Mean ISI scores decreased from 22.6 (SD, 3.4) to 16.2 (SD, 6.5), a significant improvement (p < .001). The proportion with severe insomnia declined from 68.2% to 27.3% (p = .015). PCL-5 scores improved from 55.3 (SD, 9.5) to 47.0 (SD, 12.9; p = .002), with the greatest effects in hyperarousal and intrusion domains. Physiological stress decreased from 51.2 (SD, 17.4) to 33.9 (SD, 12.9; p < .001).

**Conclusions:**

This feasibility study suggests that a personalized digital vibroacoustic intervention may reduce insomnia and PTSD symptoms in adults with PTSD. Larger randomized controlled trials are needed to confirm efficacy and establish durability.

**Disclosure of Interest:**

None Declared

